# The Body Can Balance the Score: Using a Somatic Self-Care Intervention to Support Well-Being and Promote Healing

**DOI:** 10.3390/healthcare13111258

**Published:** 2025-05-26

**Authors:** William Chance Nicholson, Michael Sapp, Elaine Miller Karas, Ingrid Margaret Duva, Linda Grabbe

**Affiliations:** 1 Nell Hodgson Woodruff School of Nursing, Emory University, Atlanta, GA 30322, USAlgrabbe@emory.edu (L.G.); 2 Trauma Resource Institute, Claremont, CA 91711, USA; msapp@communitytri.com (M.S.); emillerkaras@communitytri.com (E.M.K.)

**Keywords:** trauma, adverse childhood experiences, well-being, self-care, mental health, prevention, resiliency, interoception

## Abstract

Natural and human-made disasters, community violence, climate change, and political instability engender mental health problems worldwide. Childhood traumas, now recognized as commonplace and global in nature, augment the urgent need for mental health interventions that are accessible and scalable. The World Health Organization has called for innovative strategies that extend beyond traditional cognitive approaches. Biologically based methods are gaining recognition for their significant role in affect regulation and wellness promotion. This paper explores the potential for interventions focusing on interoceptive awareness, or noticing sensations arising from the body, to address mental health challenges, especially relevant for populations affected by trauma. The Community Resiliency Model (CRM)^®^, a low-intensity, body-based intervention that cultivates interoceptive awareness, is described and compared to other well-being interventions. Available research studies, program evaluations and anecdotal reports are presented in addition to CRM’s biological and theoretical underpinnings. The neurobiology of trauma, interoception research, and the concept of neural synchrony are briefly introduced, further explaining the likely mechanism of action and an underlying rationale for the reported improvements in well-being and resilience among individuals and communities who learn CRM body awareness techniques. Given increasing global demand and limited access to conventional mental health services, CRM and the six core skills that are taught in this model offer a promising, transferable, self-care strategy. Community dissemination has the potential to expand access in underserved populations. This review concludes by suggesting future research directions, such as the exploration of biophysical outcomes, intra- and interpersonal synchrony, and evaluation of interoceptive training for emotional regulation and populations affected by trauma or violence.

## 1. Introduction

### 1.1. Increased Need for Mental Health Interventions

Adverse childhood experiences (ACEs) are potentially traumatic events that mark the human experience. ACEs persist despite extensive research since the landmark ACE study by the Centers for Disease Control and Prevention and Kaiser Permanente [1,2]; this body of research demonstrated the considerable downstream effect of ACEs on individuals and society. The associations between childhood trauma and substance use, mental health problems, justice involvement, and many other social problems are well established. Much of the general population has experienced at least one ACE, and at least 15% have experienced multiple ACEs. ACE researchers identified a dose–response relationship of ACEs to multiple physical and chronic illnesses, including mental illness, diabetes, heart and lung disease, and cancer, and social and behavioral issues. The COVID pandemic and the ever-increasing incidence of community violence and traumatic environmental events add to the emotional stress of the general population [3,4,5]. These events can amplify the impact of childhood trauma and add to its burden on life-long health.

Responses to traumatic events often become ingrained in the mind–body, and lead to maladaptive patterns of thinking and feeling [6,7]. This is related to autonomic activity or patterns in the nervous system that are hard to counteract and that interfere with the ability to enjoy life fully. Thus, a person with toxic childhood experiences or adult trauma may helplessly continue to feel on edge or disconnected and unable to be their “best self”. The ACE studies explained the impact and mechanisms of ACEs, engendering a surge of programs for trauma-informed care and practices. The spread of ACE awareness created a considerable shift in the understanding of many problematic human behaviors (violence, substance use, self-harm, smoking, obesity), and helped to explain and de-pathologize them. Given that much of the general population has experienced childhood trauma and that many have even had multiple ACEs, an argument may be made for widely accessible mental wellness strategies to enhance the resiliency of trauma survivors.

### 1.2. Understanding Body-Based Approaches to Mental Health

Bessel van der Kolk’s book, The Body Keeps the Score, makes a clear call for body-based approaches to prevent or heal from trauma and its sequelae of anxiety, depression, somatization, dissociation, suicidality, and post-traumatic stress disorder (PTSD) [8]. He recommends “bottom-up” (or body-based) psychotherapy in addition to “top-down” therapy (cognitive talk processing). Body psychotherapy has long been available as an alternative or addition to cognitive approaches. It has been well-established for the treatment of trauma in some parts of the world for decades; however, its research base is limited [9]. Cognitive Behavior Therapy, which has been manualized, is far better researched and remains the dominant mode of therapy globally, in spite of the fact that it is not best suited for trauma processing [10,11].

Body-based alternative approaches in psychotherapy recognize the inseparability of mind–body–spirit and view perception and integration of sensory stimuli (usually experienced at an unconscious level) as foundational to our emotions and thoughts [12]. Sensations are channeled through the lower brain centers of the brainstem and limbic systems, centers that are devoid of language and integrated at a conscious level, when we can verbalize them [13]. All emotions are embodied, and sensations are experienced at conscious or unconscious levels before we are aware of our emotions [14,15]. But the body is the place where emotions are perceived [16], and body psychotherapists work in this realm. For example, in therapy sessions, clients and therapists alike may practice awareness of body sensations as a therapeutic tool for self-regulation and flexibility [13,17]. Body awareness dissolves the brain–body divide and is also used in psychotherapy to access and interpret unconscious experience [18]. Sustained attention to somatic signals affords the uncovering of the unconscious and is thus used by therapists as a tool to process trauma.

The term “felt-sense” has been used to describe the “unclear edge” or vague sense of meaning that can come from body awareness [19]. When the body’s internal state is noticed consciously, cognition is involved, and there are bidirectional brain–body messaging or oscillations [20,21]. Interoception is the scientific term for the awareness of body sensations [22] and exteroception refers to noticing sensations in or from the environment. Alertness to sensations, via the five senses or from within the body itself, provides stimulation to the brain; brain stimulation allows for focused attention and synchronicity of brain waves [13]. This attention or focus opens individuals to see themselves, others, and the environment (SOE) from a new perspective and may elicit new understandings and changes in behavior [23].

### 1.3. Significance of Examining Somatic Interventions

Body psychotherapy (also called somatic psychology) models for trauma include Somatic Experiencing, which uses the perception of body sensations to treat symptoms of PTSD and other trauma-related problems [24,25,26]. Other biologically based, trauma-focused psychotherapy models include Heller and LaPierre’s NeuroAffective Relational Model [27], Ogden’s Sensorimotor Psychotherapy [28,29,30], Rothschild’s Somatic Trauma Therapy [31,32,33], Miller-Karas’ Trauma Resiliency Model [34,35], and Steele, Boon, and van der Hart’s integrative therapy for complex trauma and structural dissociation [36], among others.

All the above models teach body-based self-regulation skills to enable the processing of the trauma by changing the interoceptive and proprioceptive sensations associated with the trauma. These models are useful across multiple cultures due to their body focus [37]. It has been suggested that misconstrued body sensations may be in part responsible for much psychopathology. Contemplative practices have been put forth to attenuate errors of interoception, restoring a person’s sense of presence and agency [38], and such self-care practices may be helpful for persons with or without psychopathology. Thus, recent developments in interoceptive awareness training for self-care are important.

Interoceptive awareness skills, which can be taught in a brief session, offer accessible tools for both preventative and acute mental health support. Recent scholarship highlights interoception—the perception of internal bodily sensations—as a promising body-based strategy for individuals affected by trauma [8]. This review advances that perspective by examining the Community Resiliency Model (CRM)^®^ as an illustrative example. Its theoretical foundations and physiological mechanisms are explored, and current evidence—including empirical studies, implementation reports, and anecdotal accounts—is synthesized. The paper also identifies knowledge gaps and evaluates CRM’s potential as a scalable, body-based mental health intervention.

## 2. Theoretical Bases for Interoceptive Awareness Based Interventions

### 2.1. Fight and Flight

In 1915, Walter Cannon first introduced the fight and flight response concept, describing how the autonomic nervous system, in conjunction with the release of adrenaline, readies an animal for an emergency reaction, commonly called “fight or flight” [39]. Cannon’s groundbreaking work elucidated how this response triggers alterations in blood flow, effectively marshaling the body’s resources to enable what he termed a “violent display of energy”. In response to a perceived threat, cortisol and adrenaline are released, and the person feels a surge of energy to respond to the threat by fighting or fleeing.

The “tend and befriend” response concept explains that within certain contexts, “tending and befriending” occurs when people are faced with a threat. Taylor found that individuals who identify as female release oxytocin as part of their stress response, which may override the fight/flight response and can lead individuals to prioritize nurturing, tending to children, and forming alliances with other women for the protection and safety of the group [40,41]. It can also result in attempts to tend and befriend the aggressor, which may increase likelihood of survival. When an aggressor encounters a person in this state, the surge of oxytocin may also trigger the release of oxytocin in the aggressor (i.e., through mirror neurons), potentially increasing the woman and her social group’s chances of survival. Even though Taylor’s research was on people who identified as female, nothing would preclude people of any gender identification from using this strategy (e.g., first-responders rushing into a burning building to rescue trapped co-workers, etc.).

When an existential threat looms, and a person cannot engage in tend and befriend, fight, or flee, the freeze response, also known as “tonic immobility”, may come into play. This response is characterized by profound motor inhibition and is evoked by inescapable threats across various species, including humans. During predatory encounters, some animals may opt to freeze or simulate death as a survival strategy. Tonic immobility becomes the preferred course of action when the organism perceives little immediate opportunity for fight or flight [42]. Note that stress experienced in everyday life (e.g., a dispute with a co-worker or customer, a conflict with a family member) can cause us to leave the Resilient Zone and we may have less access to higher brain reasoning. These are far from existential threats, but we may experience threat responses, especially those of us who are hypervigilant from previous trauma experiences.

The intricate design of the human nervous system is responsible for triggering these survival responses in the presence of perceived existential threats. CRM training helps individuals understand the diverse array of multi-sensory triggers that can activate when reminded of a traumatic experience or highly stressful event. Van der Hart, et al., described these triggers as encompassing sensory data, time-related stimuli, routine daily life occurrences, events occurring during therapeutic sessions, emotional and physiological states, stimuli evoking memories of intimidation by perpetrators, and ongoing traumatic experiences [43].

Nearly anything can serve as a multi-sensory reminder of a traumatic event. By grasping the concept of multi-sensory cues, individuals gain insight into the biological underpinnings of their sensory responses. This enhanced understanding fosters a more comprehensive awareness of the automatic nature of their survival responses. Through interoceptive awareness, individuals can develop greater awareness of the multi-sensory triggers to intervene with a sensory awareness, helping them return to a more balanced state, a resilient zone.

### 2.2. The Stress Response

The mechanisms underlying the efficacy of the intentional interoception of CRM are yet to be thoroughly explored. However, emerging research in stress physiology and somatic interventions suggests a direct influence on sensory processing systems. During periods of stress, the brain aims to (1) discern the stressor’s origin, (2) appraise its threat potential, (3) deploy a behavioral response (e.g., fight, flight, freeze, tend and befriend), (4) mitigate the stressor’s physiological impact, and (5) encode and/or learn from this experience for the future. While salient stimuli can be processed for event learning during acute-stress states, more nuanced learning tends to occur once the actual or perceived threat is resolved. Importantly, threat resolution increases a sense of safety permitting cognitive assimilation of information in more contextual detail and promoting adaptive strategies for the future [44].

In acute stress situations, the brain’s automatic default mode curtails higher executive functions in favor of primal motor- and emotion-driven reactions. This feature of brain function prioritizes threat detection to optimize survival. This mechanism is a protective, evolutionary-entrenched negativity bias. Executive control mechanisms (or top-down processes) are modulated dynamically, reallocating metabolic resources to fundamental stress-response centers. This translates to an augmentation of bottom-up control, facilitating prompt physiological responses [45]. “Bottom-up” control allows primal survival responses to override “top-down” processes—an elegant design that increases the likelihood of survival in acute situations where “thinking about the best course of action” rather than just “acting” may result in our demise.

Although evolutionarily adaptive across a spectrum of acute experiences, chronic activation of this stress response impedes experiential learning; because of this, there tends to be a fixed response to future stressors of a similar nature or magnitude [46]. If a given survival strategy aided in our survival in the past, it would seem advantageous to use the same strategy when faced with similar situations in the future. However, such fixed responses can result in a type of survival strategy (hyper-regularization), in which we perceive every “match as a forest fire” and respond with high magnitude to a stressor that may not warrant such a response. CRM theory proposes the need for interventions at this juncture via an array of skills designed to interrupt this fixed response, potentially counteracting unwarranted or exaggerated stress reactions [47]. Moreover, CRM education provides learners with a nuanced understanding of their stress physiology and responses during distressing events (e.g., trauma). This allows for a shift towards both physiological and psychological empowerment related to better controlling oneself when under stress.

Within a psychosocial frame, most sources of stress originate from the self, other persons, and environmental (SOE system) threats. The more difficult or psychologically complex the stress, the more overwhelmed we become, and the more likely we are to be pushed out of the Resilient Zone. This leaves us vulnerable to becoming “stuck” in a dysregulated high- or low-arousal state. Ultimately, if this dysregulated physiological stress cycle persists, there is an increased risk of chronic physical and mental health disorders, as has been well documented as consequences of ACEs [6]. Visceral interoception (i.e., sensing activity of heart, lungs, and gut) is associated with the ability to access the generative results of sensory processing in the form of emotional feeling, intensity, and discernment. 

Conventional cognitive interventions offer stress modulation via predominantly top-down processes. A top-down approach is not ideal during acute stress processing. In contrast, bottom-up strategies emphasize the amplification of sensory-based safety cues within the SOE system. CRM techniques facilitate an immediate re-assessment of these sensory cues that could help optimize the balance between bottom-up and top-down processes during stressful encounters. Consequently, CRM may offer a variety of ways to intercept maladaptive stress reactions, pivoting attention from inherently self-referential, stress-induced cognitions towards a more congruent perception of one’s position within the SOE milieu [48].

### 2.3. Interoception

A deeper look at interoception—the internal sensing of physiological states—is important to understand how a simple sensory awareness toolset works. CRM emphasizes body sensations, suggesting a synchronized interplay between the brain’s central and autonomic pathways. Such coordination underlies key facets of resilience, including but not limited to self-control, attachment, and social engagement [23,49]. Khalsa and colleagues [50,51] metaphorically describe interoception as the brain’s “tightrope act”, coordinating bodily perceptions into a sensible interpretation of our surroundings. Targeted interoception, as taught in CRM, appears to augment wellness by enhancing or perhaps remodulating the brain’s existing regulatory mechanisms. 

The multifaceted nature of interoception incorporates a constellation of physiological systems, from cardiovascular to gastrointestinal domains, whose integration is critical to how we perceive and interact within changing environments [52]. This integration promotes the processing of intrinsic and extrinsic sensory signals to the body. These signals interact within specific neural conduits, such as vagus nerve and spinal tracts, to communicate across central-autonomic pathways [53]. Central processing structures, such as the insula, anterior cingulate, and prefrontal cortices, are implicated in decoding and contextualizing these signals, thereby influencing an individual’s conscious perception of bodily states [54].

**Figure 1:**
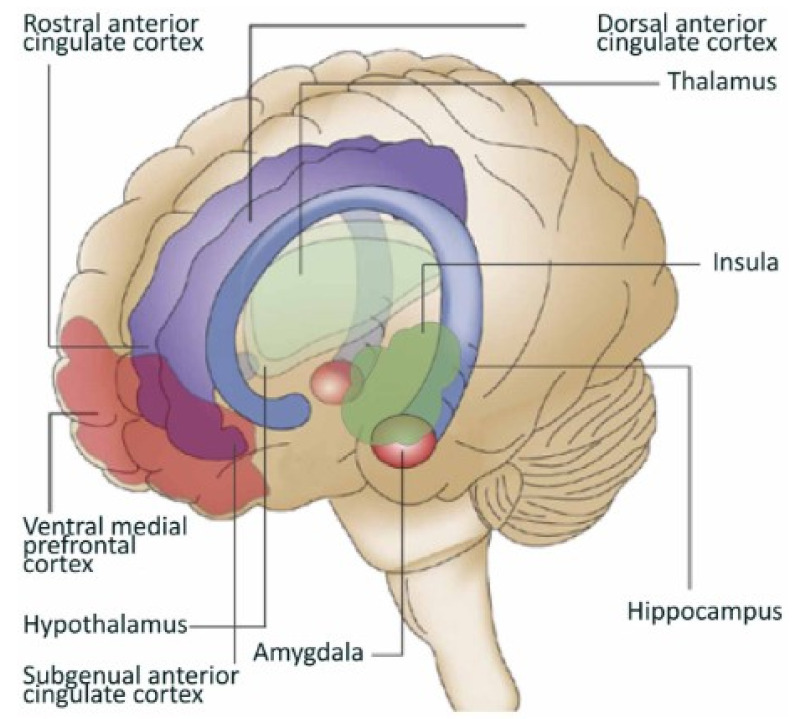
Brain regions involved in resilience to stress adapted by [56].

Specifically, the insula is the critical structure in this network that helps us make sense of our world, acting as a hub that monitors the body’s physiology and connects with the frontal cortex to coordinate a balance between the order and entropy (randomness) of our stress response systems (Figure 1). Interoception’s central role in modulating homeostasis and allostasis also extends its influence on cognitive and affective regulation [55]. The body perpetually engages in interoceptive processes at both conscious and subconscious levels, supplying the individual with a stream of internal physiological feedback. This feedback mechanism—which the CRM skill of Tracking (i.e., intentional interoception) enhances—is instrumental in facilitating adaptive responses to a variety of stimuli, such as thermoregulation and nociceptive (i.e., pain) perception.

## 3. Interoceptive Awareness Training

### 3.1. Applications and Measurement

Aside from the use of body awareness (interoception) for psychotherapy purposes, training in body awareness techniques for a variety of populations is currently being researched. There has been an exponential growth in interest in interoception in the neurobiology and psychiatric literature. This has led to international gatherings of experts from diverse disciplines, and the development of a “roadmap” for future research [50]. Interoception has even come to the attention of lay audiences [57]. In psychiatry, it has been suggested that unregulated depression and anxiety states involve disruptions to interoception and self-referential interoception belief states [56]. Interoceptive processing, which has been categorized into accuracy and sensibility components, is currently the topic of a wealth of research.

A significant contribution to the field of interoception has been the development of the Multidimensional Assessment of Interoceptive Awareness-2 scale (MAIA-2). The measure has eight subscales: 1—Noticing (Awareness of Body Sensations), 2—Not-Distracting (Emotional Reaction and Attentional Response to Sensations), 3—Not-Worrying (Emotional Reaction and Attentional Response to Sensations), 4—Attention Regulation (Capacity to Regulate Attention), 5—Emotional Awareness (Awareness of Mind–Body Integration), 6—Self-Regulation (Awareness of Mind–Body Integration), 7—Body Listening (Awareness of Mind–Body Integration), and 8—Trust (Trusting Body Sensations) [58]. This scale has been widely used in research studies with a variety of applications.

Concurrent research in the field of interoception includes (1) understanding the neurobiology of interception and (2) research on short-term interoceptive awareness interventions for clinical populations. Short-term interoception trainings do not aim to provide psychotherapy, but they constitute a therapeutic intervention. For example,

- A 7-week mindful self-regulation-based interoceptive awareness training for a small group of special education students found significant correlations between interoceptive awareness and emotional regulation, and improvements in both measures [59,60].
- A 10 to 12 h online training program of interoceptive and emotional awareness, mindfulness, emotion recognition, and emotion regulation strategies found reduced emotion suppression and greater impulse control relative to placebo [61].
- A 4-session online interoceptive training intervention (Reconnecting to Internal Sensations and Experiences) increased interoceptive sensibility with the goal of reducing suicidality in veterans [62].
- An 8-week manualized program in Mindful Awareness in Body-oriented Therapy used interoception as an adjunct to buprenorphine for a small group of individuals with opioid use disorder, and results included satisfaction with the training and increased interoceptive awareness [63].
- A study of a 4-day training in Emotional Freedom Technique (EFT) for clinical therapy clients found significant improvements in resting heart rate, blood pressure, and cortisol, as well as several psychological measures [64]. EFT blends cognitive and exposure techniques with somatic stimulation of acupressure points on the face, arms, and head [64,65].

These trainings that entail interoception components are demonstrating effectiveness for specific clinical populations. Interoception is also at the crux of some innovative interventions for mental health pathology, for example,

- Weng and colleagues are suggesting that interoceptive pathways can be manipulated through neuromodulation of the vagus nerve, slow breathing, and mindfulness-based interventions at neural, behavioral, and psychological levels to alter interoceptive signals to improve functioning and adaptive behavior [17].
- Auricular vagus nerve stimulation is being used as neuromodulation of the vagus nerve areas to alter emotional processing with interoceptive functioning in emotional disorders [66].

### 3.2. Training Specific to Wellness Programs

Increasingly popular programs that embrace elements of body awareness (or interoception) are mindfulness, Dialectical Behavior Therapy (DBT) Skills Training, yoga, Tai Chi, meditation, and other self-care modalities; these modalities are reaching broad groups of people, and they may confer considerable benefits for well-being. Many of these trainings or practices include content other than developing and using body awareness; however, the teaching of interoceptive awareness techniques and sensory-perceptive states by itself has been recognized as salutogenic [18]. Salutogenic modalities are asset- or strengths-based (vs. deficit/disorder-based) and focus on improving health, well-being, and sense of coherence, as well as managing stressors and enhancing the meaningfulness of life [67].

One of the most significant contributions in salutogenic wellness promotion is the mindfulness programs that have sprung up widely and are currently being offered to persons in high-stress occupations and to the general public. Mindfulness has been described as a whole-body experience, rather than just a mental state.

The term “mindfulness” is a translation of a Buddhist word meaning awareness, alertness, and insight cultivated by meditation or focused attention; the goal of mindfulness practice is happiness and understanding the nature of one’s existence [68]. Mindfulness involves the in-the-moment, non-judgmental awareness of thoughts, emotions, and sensations. This type of focused attention allows for a cognitive reset and openness to change. Increasing attention is also being given to narrowing the definition of interoception by examining the neurobiological relationships with mindfulness and other alternative modalities. These ongoing efforts are critical for conceptualizing similarities and distinctions across these programs by identifying key brain hubs and/or networks involved when engaged in activities such as mindfulness and meditation, among others [69]. Mindfulness programs have grown in number, and for the most part have been presented in a cognitive-behavioral format since being first introduced by Jon Kabat-Zinn [70]. That the mindfulness–interoception connection has been made is evidenced by the 10-fold increase in scholarly articles in Medline (between 2011 (n = 5) and 2023 (n = 51)).

There is no doubt that mindfulness and contemplative practices such as meditation and Tai Chi can impact mental health and reduce stress. Another mindfulness program embedded in Cognitive Behavior Therapy is Dialectical Behavior Therapy (DBT) Skills Training, which is a 3-month self-care approach that incorporates mindfulness and some awareness of body sensations [71]. CRM is an interoceptive awareness training in the category of short-term interventions; this modality will be described in detail in this paper as an exemplar to reduce responses to trauma and improve mental well-being [34,35]. Compared to the above examples, CRM is more quickly taught and easy to disseminate because it can be shared or taught by non-health professionals. Because of these features, CRM can help bridge treatment gaps, especially in poorly resourced communities and countries with healthcare inequities. Note is made here that all five authors of this paper are broadly experienced CRM teachers. Additionally, one has taught DBT Skills Trainings and a CBT-based meditation and mindfulness program [72].

## 4. The Community Resiliency Model (CRM) ^®^ as an Exemplar

### 4.1. Origin of the Model

The Community Resiliency Model (CRM)^®^ is a novel mental wellness training focused on interoceptive awareness that has a growing evidence base. CRM emerged from work with disaster survivors and seems to confer a more compassionate understanding of our own mental health and that of other people. The model is intended for self-care or self-help but is also for people who work in the service of others [34,35]. Understanding the global occurrence of trauma, and recognizing the need for easily accessed, sustainable mental health interventions, Elaine Miller-Karas and Geneie Everett co-created Trauma First Aide in 2004, as a short-term intervention, influenced by Peter Levine’s Somatic Experiencing^®^ (SE), Jean Ayres’ Sensory Integration Theory, Eugene Gendlin’s Focusing, Lamaze childbirth education, and their knowledge of the biology of trauma.

Trauma First Aide engendered CRM, which is now a stand-alone, self-care model consisting of six emotion stabilization skills: Tracking, Resourcing, Grounding, Gesturing, Help Now!, and Shift and Stay (Table 1). Each CRM skills hinges on the ability to use interoceptive or body sensation awareness, which is practiced through the main skill called “Tracking”. Once this initial core skill is learned, the five other skills are beneficial, providing alternate opportunities to settle the nervous system in the moment, benefiting from the focus on sensation awareness.

A sister model to CRM is the Trauma Resiliency Model (TRM). Unlike CRM, which is designed to be used by the community, TRM is for behavioral health practitioners’ use in psychotherapy for clients who have experienced trauma. TRM’s ten skills include the six self-help wellness skills of CRM, all designed to bring the nervous system back into a more balanced state. The extra skills were adapted from other somatic interventions and are used to reprocess the biological experience of threat and fear. These skills complete the survival responses thwarted at the time of the traumatic event in present-moment awareness. The three adapted skills are called Pendulation, Titration, and Completion of Survival Responses. While valuable, these go beyond interoception and self-care, are not transferrable, and are not part of CRM.

### 4.2. Theoretical Underpinnings

The Community Resiliency Model (CRM) is informed by neuroscience, somatic psychology, and public health. Its theoretical foundations reflect a comprehensive, biologically based approach to trauma healing and wellness promotion. Key concepts are:

- Nervous System Regulation: CRM highlights the role of the autonomic nervous system in shaping how individuals respond to stress and safety. Drawing from Polyvagal Theory, CRM emphasizes the vagus nerve’s role in regulating emotional and physiological states and supports returning to a “Resilient Zone” where optimal functioning occurs.
- Neuroplasticity: CRM is grounded in the principle that the brain and nervous system can reorganize through experience. Repeated use of CRM’s wellness skills supports the development of new neural pathways that enhance emotional regulation and resilience.
- Solution-Focused Psychotherapy: CRM reflects the strengths-based orientation of this short-term therapeutic model, focusing on clients’ internal resources and envisioning a preferred future. It operates on the belief that individuals possess the capacity for change and that small, attainable steps can lead to meaningful progress.
- Sensory Integration Theory: CRM recognizes the importance of effective processing of sensory input—such as touch, movement, and sound—for behavioral and emotional regulation. When sensory integration is disrupted, individuals may become over- or under-responsive to stimuli. CRM helps restore balance by supporting awareness of internal sensory experiences.
- Somatic Psychology: CRM incorporates bottom-up approaches to healing, prioritizing body awareness over cognitive processing. Individuals are guided to track sensations—especially those linked to well-being—which supports nervous system stabilization and promotes embodied resilience.
- Public Health and Social Justice Orientation: Designed as a scalable, community-accessible model, CRM can be used by both professionals and laypersons. It is grounded in equity and cultural humility, with a focus on reaching underserved, trauma-impacted populations and fostering collective resilience.
- Systems Thinking and Community Psychology: CRM acknowledges that trauma and healing occur within relational and societal contexts. It promotes a systems-based approach to recovery, emphasizing empowerment, mutual support, and sustainable community capacity-building.

### 4.3. CRM’s Cornerstone Concept

**Figure 2:**
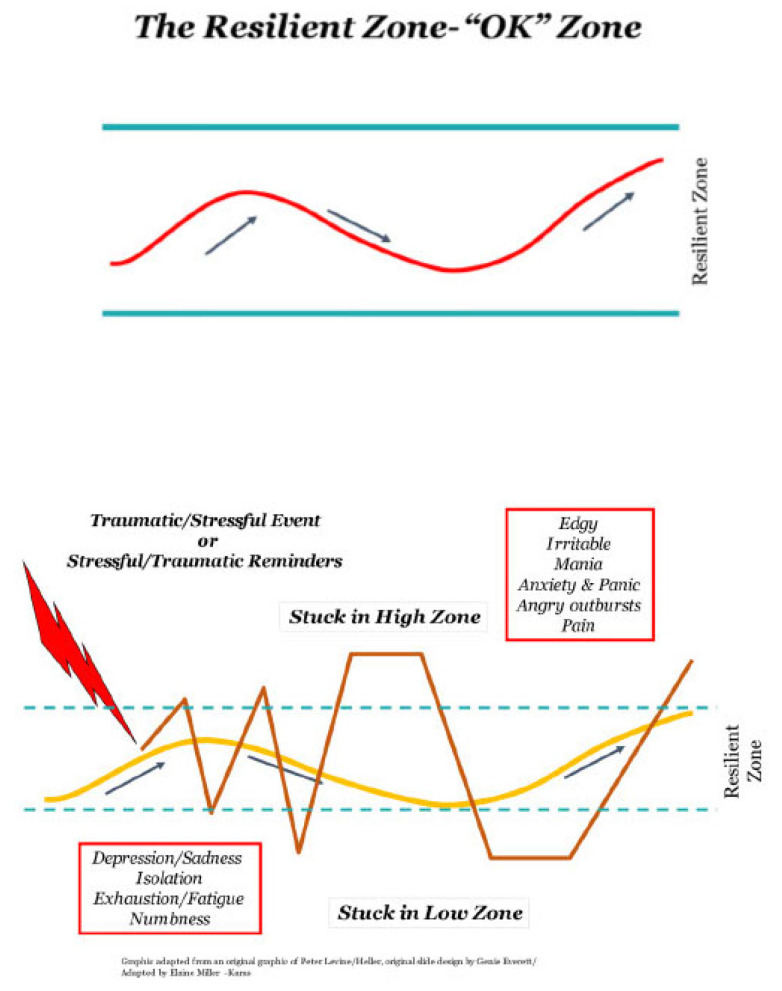
The Resilient Zone—regulation to dysregulation. When in the zone, we may move up and down but are still functioning as our best selves. When major trauma occurs or small stressors over time add up, we may be bumped out of our zone. It is okay to bump out if we don’t get stuck outside our resiliency zone.

The foundational concept of CRM is the “Resilient Zone” (one’s bandwidth of embodied well-being) and the High and Low Zones (states of hyper- and hypo-autonomic arousal) (Figure 2). The goal of CRM is to widen this bandwidth and to have the capacity to return to the Zone when in an emotionally dysregulated (Low or High) state. Other key concepts conveyed in trainings include sources of emotional dysregulation, ACEs, common responses to stress and trauma, the autonomic nervous system, the coordination of higher and lower brain centers, and post-traumatic growth. The CRM intervention incorporates education on the automatic nature of survival responses, encompassing fight, flight, freeze, and “tend and befriend” reactions. This educational component may mitigate the pervasive feelings of shame and self-blame that often accompany individuals’ automatic survival responses to existential or even everyday threats or insults.

### 4.4. Comparison of the Resilient Zone to Related Concepts

The Resilient Zone concept may be compared with Wilbarger’s “Optimal Arousal Zone” [74,75] and Ogdon and Minton’s “Modulation Model” [28], which use Daniel Siegel’s framework of a “Window of Tolerance”, an area of arousal in which we each can function adequately [76]. The “Window” pertains specifically to any moment when we have more tolerance for some emotions and situations than others. Being outside of the window is chaos, with one end of the window characterized by hyper-arousal and the other end by hypo-arousal. Similarly, Brier’s model is used as a framework for psychotherapists to assist in case conceptualization [77]. In this model, Level One is “Numb and incongruent”; Level Two is “Congruent and controlled”; and Level Three is “Relived traumatic intensity”. In comparison to CRM, Briere’s Level One would be comparable to CRM’s Low Zone, Level Two to the Resilient Zone, and Level Three to the High Zone.

A challenge with the semantics of the “Window of Tolerance” is with the definition of tolerance. In the Oxford dictionary, tolerance is defined as the capacity to endure continued subjection to something or someone and the ability or willingness to tolerate something. The Resilient Zone, also called the Zone of Well-Being, is a dynamic condition of vitality existing in all human beings. This vitality includes a rhythm of energy where human emotions like sorrow, anger, joy, or happiness can exist with their associated sensations.

Behaviors connected to the Resilient Zone can include dynamic advocacy, such as when a person is angry about a world condition or organic sadness when someone participates in a tradition remembering a beloved family member who has died. The accompanying anger or sadness in these examples is not “tolerated” but rather experienced within our Resilient Zone without judgment and without feeling overwhelmed by their intensity. CRM’s Resilient Zone is not about tolerance but about embracing embodied well-being. When in our Resilient Zone, our sensations, emotions, and thoughts are well integrated. This conceptualization also lays the foundation for how interoceptive awareness is critical for stabilizing the nervous system. The theoretical basis and evidence for CRM, an example of interoceptive awareness training, is described in this article.

Another challenge is that interventions that use the Window of Tolerance framework describe traumatic states to help mental health providers conceptualize their clients’ experience in one-on-one therapeutic environments, rather than equipping the client with concepts that can help them in their own self-care. One of the values of the Resilient Zone concept of CRM is that it provides the client/patient with a positive conceptualization that they can use themselves to help make sense of their own experience without pathologizing it. Rather than focusing on problems or deficits, the positively named Resilient Zone is a state where we can think clearly and work with others even though we may be irritable, angry, sad, lonely, or upset, i.e., we can function adequately. We do not need to be happy or calm to be in the Zone.

In CRM training, accessible language is used (and may be adapted to accommodate any group, including children) to describe the human experience, not only in relation to trauma but also in daily life when experiences can push any amongst us into a hyper-aroused state (High) or a hypo-aroused state (Low). Another interpretation of the Resilient Zone might be at an organizational level, where employees are stressed and anxious (High Zone) or apathetic and fatigued (Low Zone). Learners of CRM are encouraged to create their own name for the Resilient Zone; the Spanish translation means the “Zone of Well-Being”, and other names that have been coined are the OK/Swag/Reset Zone.

The Zones are explained to make these states more understandable, not only to mental health providers but also to any group, organization, or community member. The model is taught by natural leaders or peers, not only by mental health providers. The concepts of the different Zones in CRM, especially the use of the term “Resilient Zone” or “Zone of Well-being”, enables anyone—whether they are receiving therapy services or not—to understand how they are functioning at any given moment, to be able to assess in real time how stress is affecting their well-being, and then to implement CRM skills to improve their mental state, as is necessary.

### 4.5. CRM Training Compared to Mindfulness

Mindfulness training is a potent form of wellness practice. CRM training is significantly different from mindfulness training in several ways, although each involve interoception, and have been used in conjunction with each other. Mindfulness training is usually taught in a Cognitive Behavioral framework, which often requires eight sessions, but its origins come from ancient Eastern traditions, including meditation; in contrast, CRM emerged from physiologically and biologically oriented, trauma-centered influences, including body psychotherapy. Mindfulness does integrate body awareness, often with a focus on the breath or a mantra repeated to oneself, whereas CRM teachers scrupulously avoid directing attention to the breath due to its potential as a “trigger”; attention to the breath is only done in the context of a learner describing a source of strength, and spontaneously taking a deep breath, a sign of moving into the Resilient Zone. CRM was designed initially for trauma survivors, and although it is now being taught to many people who are not survivors of trauma, the ACE- and trauma-informed nature of CRM, and its assumption that all learners are likely to have experienced trauma, means that it is exquisitely trauma-sensitive. Because body awareness might cause arousal or flooding in persons with trauma histories, cautions or caveats for practicing the skills are made throughout the training.

Mindfulness entails “the awareness that emerges through paying attention, on purpose, in the present moment, and non-judgmentally to the unfolding of experience moment by moment” [78] (p. 145). This may include being in a quiet place, sometimes with a single point of focus, like the breath. Conversely, CRM can be used both at random moments (just paying attention to sensations in the moment) and under dire or unpleasant circumstances (e.g., by intentionally touching one’s clothing or skin). While emotions and thoughts are addressed, they are not the primary purpose of the intervention; hence, there is no need for a non-judgmental attitude. Rather, there is simply intentional attention to sensations.

Once sensations are noticed, they might be localized, characterized, and even named (both the nature of the sensation and the emotion), but by then, CRM will have done its magic—engagement of the pre-frontal cortex with lower brain networks may serve to bring the brain back into “synch”. Further, there is no single point of focus, but rather, a menu of ways to engage in interoceptive awareness to select from, depending on the situation. Thus, Tracking, or another CRM skill, can be a direct route to stability and emotional balance. And, while mindfulness and meditation or other wellness practices will most certainly widen the bandwidth of well-being (the Resilient Zone), the techniques may be less valuable for returning to the Zone when pushed out. Note may be made that one of the current authors taught a Yale University 8-session mindfulness and meditation program to 90 unhoused American youth who were at-risk for addiction, with highly significant results on multiple well-being parameters [72].

CRM classes universally include several key concepts which are not generally associated with mindfulness training:

(1) The Resilient Zone concept provides a framework for understanding regulated and dysregulated states of mind.
(2) Body sensations are linked to common pleasant and unpleasant emotions using body map slides [14,15].
(3) Common emotion-dysregulation responses to stress and trauma (behavioral, cognitive, emotional, relational, spiritual, and physical) are discussed in an interactive format, even virtually.
(4) ACEs are touched on, along with post-traumatic growth and/or positive experiences of childhood [79].
(5) Brain networks related to survival (brainstem), emotions (limbic), and cognition (cortical) areas are explained in simple terms and tied into the Resilient Zone and dysregulated (High and Low) states so that learners have a new and more compassionate understanding of their own personal experiences, as well as the behaviors of people around them.

### 4.6. When Interoceptive Awareness Is Activating: The CRM Teacher Response

Teaching people interoceptive awareness skills can cause many reactions—pleasant, unpleasant, or neutral. Safety is paramount in how CRM is shared, and CRM teachers are trained to be trauma-sensitive and vigilant. A key CRM concept is learning to distinguish between sensations of distress and sensations of well-being. Once that awareness is developed, learners understand that they can shift their attention; they have a choice of what sensations to pay attention to—those of well-being or those of distress. CRM is a strength-based approach and, as such, at the beginning of a CRM session, learners are invited to notice sensations connected to a personal resource (often family/friend, their faith, a certain place/belief). This way of teaching reduces the likelihood of activation. Several of the skills (e.g., Grounding, Tracking) could be activating and retraumatizing, especially for trauma survivors, and mention of this is made at various points of the training. It is also made clear upfront that paying attention to sensations can be novel and unfamiliar and, for some, distressing. Thus, any time the learner does not want to try a skill, they can stop.

Invitational language integrated into the model is essential; CRM teachers are instructed that learning the skills and interoceptive awareness is always a choice. To create greater safety, CRM teachers are prepared to use Help Now! strategies. These strategies can help a person return to present-moment awareness and reduce or eliminate sensations that are distressing. Note that CRM teachers do not recommend any pattern or practice of breathing. This is because noticing the breath can itself trigger an unpleasant reaction. However, when Resourcing, learners often do take a spontaneous deep breath and the teacher may remark on that deeper breath because it signals a resetting of the nervous system, strengthening the Resource itself.

For some learners having difficulty with interoceptive awareness, CRM teachers know that working outside the body first can be beneficial. For example, a person can be invited to touch items with different textures and describe whether they are soft, rough, smooth, etc., and inquire whether touching a particular surface is more pleasant or not. However, CRM is always invitational. There are some people that do not want to learn about interoceptive awareness, and their choices are honored. Sometimes, by engaging in education and in gentle guidance and invitation, CRM skills may be introduced. This gentle approach has also been used for people with chronic pain, and they report a reduction in symptoms. Current studies are being conducted on CRM in Sickle Cell Units at Loma Linda University in Loma Linda, California, and Grady Hospital in Atlanta, Georgia. Although psychosis comes to mind as a contraindication, CRM’s Help Now! and Resourcing skills have been used successfully with persons in psychotic states to reduce activation. In this situation, as always when sharing CRM skills, invitational language is paramount. This model is not contraindicated for persons with a trauma history (rather, it was developed with those persons in mind). CRM education includes the concept of “memory capsules” related to trauma (flashbacks); once understood, learners have new insight and tools to handle multi-sensory reminders of painful experiences. While CRM has no contraindication, persons in an inebriated state would derive little benefit. Persons with intellectual disabilities or dementia could benefit from an adapted somatic awareness training. CRM is meant to be adapted to the needs and intellectual level of the target learners. It might also be helpful to caregivers for stress reduction.

## 5. The Community Resiliency Model: Evidence Thus Far

### 5.1. Practical Application and the Evolution of CRM

Disaster settings generally preclude research opportunities, but the disaster response to the Asian tsunami of 2004 demonstrated that somatic-based skills training (Somatic Experiencing) could be effective in reducing distress of survivors. One hundred and fifty (150) survivors with trauma symptoms received 75 min of somatic training in affect modulation and self-regulation; at the 8-month follow-up, 90% of participants reported significant improvement or being completely free of PTSD symptoms [80]. In a separate study of 53 tsunami survivors, a brief Trauma First Aide intervention was associated with similar findings [81], and a Hurricane Katrina study of social workers who received the somatic regulation skills and were compared with a non-skills group demonstrated similar results [82].

Humanitarian efforts for earthquake and hurricane relief supported the effectiveness of the basic body-based self-regulation skills [82,83] and CRM emerged as a stand-alone, brief intervention to teach self-care skills. Leitch and Miller-Karas (2009) focused on a one- or two-session CRM training for frontline workers after an earthquake in China; these workers reported positive perceptions and reported use of the skills with clients and for their own self-care [83]. CRM has since been deployed internationally by the Trauma Resource Institute to assist trauma survivors (see Table 2).

It is worthwhile here to mention Mental Health First Aid (MHFA), a globally disseminated intervention. Members of the public are trained as first responders for mental health issues in their communities to help by assessing, listening, and offering support to distressed persons; however, a recent literature review of MHFA found no effects on the helpfulness of trainees’ actions on recipient mental health [84]. A coping skill as simple as noticing body sensations (as is done with Resourcing when someone shares something that helps them get through challenging times) can be easily added to the MHFA repertoire to offer a skill to emotionally distressed persons. CRM is highly adaptable and could be easily integrated into MHFA interventions. The MHFA provider can choose a CRM skill to use based on the situation, person, and their own preference. In this way, CRM does not replace, but can be used in conjunction with, MHFA. Likewise, CRM has been integrated into two Emory University mindfulness/meditation programs described elsewhere in this paper.

### 5.2. Empirical Evidence Summary

The evidence base supporting CRM’s simple body awareness skills for well-being is emerging. To date there have been three randomized controlled trials reported in the literature: two were conducted with healthcare workers and the most recent is a pilot study with pregnant patients. Each of these studies resulted in statistically significant improvements in selected psychological and socioemotional (wellness) measures, at a level of *p* ≤ 0.05, although these measures varied across studies. Effect sizes, or Cohen’s d, are also reported, indicating the impact of the CRM intervention. For clarification, Cohen classified effect sizes as small (d = 0.2–0.4), medium (d= 0.4–0.7), and large (d = 0.8 or greater), with an effect size greater than 0.2 generally considered meaningful change [85].

A randomized controlled trial (RCT) of CRM used an active control, testing a 3 h in-person CRM class against a nutrition class for nurses [86]. The findings were sorted first by statistical significance as reported, but also by clinical (practical) significance and identified by the effect size categories (small, medium, and large). Significant improvement surfaced for several measures when analyzed over time. For the CRM group, the outcomes that significantly improved were well-being (*p* = 0.006, d = 0.87), resilience (*p* = 0.004, d = 0.42), secondary traumatic stress (*p* = 0.009, d = −0.34), and somatic symptoms (physical complaints) (*p* = 0.004, d = −0.38). Eighty percent of the nurses in the CRM group showed significantly improved well-being from baseline at 1 year, demonstrating improvement may be maintained over time.

A second RCT was conducted with healthcare workers during the Delta surge of the COVID-19 pandemic [87]. This study delivered a single hour-long virtual CRM class with no active control (control group members were later invited to a CRM class). The intervention and control participants provided baseline survey data, with follow-up at 1 week and 3 months. The 50 intervention participants reported significantly higher improvement at 3 months on the well-being measures (WHO-5, *p* < 0.0087, d = 0.66), 2 items of the Warwick-Edinburgh Mental Well-Being Scale (*p* < 0.0004, d = 0.66), an adapted teamwork measure (*p* ≤ 0.0002, d = 0.41), and the Secondary Traumatic Stress Scale, (*p* = 0.0058, d = −0.46). The Multidimensional Assessment of Interoceptive Awareness-2 (MAIA-2) Noticing scale showed a significant time effect and group-by-time effect (*p* < 0.02), with a moderate effect size for the changes from baseline to 3 months (d = 0.52). 

A pilot RCT recently concluded that CRM training combined with motivational interviewing was a feasible and acceptable intervention among pregnant women at a midwestern United States federally qualified health center. At 2 months post-intervention, a statistically significant effect on health-promoting behaviors (*p* ≤ 0.05) and a large effect size (0.98) were found. Also notable was an improvement in anxiety measured at 6 months post-partum nearing significance (*p* = 0.08) and a large effect size (−0.72) [88].

In addition to these RCT findings, CRM has been associated with improvement across other health measures such as reduced hostility, depression, anxiety, and post-traumatic stress symptoms in quasi-experimental studies with a single group pre-/post-test and observational designs [82,89,90,91,92,93,94], adding significant cross-sectional results to those measures found to improve longitudinally. For example, a ½-day training of 20 indigent women in drug treatment yielded significantly improved well-being (small effect size) and reduced somatic, anger, and anxiety symptoms (with moderate to large effect sizes) in a pre–post mixed methods design study [93]. More recently, high-risk pregnant women were offered participation in a “Wellness Within” support program. Of those, 40 participated in a two-hour CRM training and responded to surveys distributed over time. The outcomes of interest—resilience, depression and well-being, and experience of discrimination—were tracked over time. CRM participation was associated with significant improvement (*p* < 0.0001) in patient-reported resiliency from pre-intervention to 1 week after the intervention, and maintenance of improved resilience through labor and birth until the six-week post-partum visit. The study results supported CRM as a sustainable and effective intervention for promoting resilience in pregnant people, particularly in the context of a high burden of discrimination [94]. Another pre-/post-test evaluation study of 22 emergency department providers did not reach significance but there were improvement trends in resiliency and professional quality of life [91].

Within the United States, published study samples consist of professionals and community members, persons with addiction, pregnant patients [87,93,94]. Outside the United States, Aréchiga (2023) and colleagues implemented a two-day CRM intervention in post-Ebola Sierra Leone for community members, resulting in significantly improved mental health measures of depression, anxiety, and PTSD symptoms (as measured by Harvard Trauma Questionnaire Revised Cambodian Version) both immediately and after six months [95]. The Resilience and Grounding Organization (RRGO) delivered a CRM training to survivors of the Tutsi genocide decades after their trauma; the findings included significantly improved depression, secondary traumatic stress, and trauma symptom ratings [92]. RRGO is in the process of expanding their CRM training to cancer patients and camp-dwelling refugees of the nearby wars/conflicts. CRM has been evaluated while blended with other interventions, such as behavior change or lifestyle [88,96]. A qualitative study of workers in a clinic for unhoused, indigenous individuals in New Mexico integrated relational–cultural theory, motivational interviewing, and CRM [97]. From this study, CRM was determined to be a tool to more effectively interact with clients with high potential for crises, such as violence or self-harm. The goal was to increase system-level support for the multi-disciplinary healthcare team and ultimately improve both healthcare worker well-being and the client’s sense of being cared for, and, for both, an increased sense of personal safety while in the clinic.

Program evaluations and qualitative inquiry are lower levels of evidence, but still build knowledge about CRM’s applicability and acceptability, for future implementation, scale up, spread, and indications for sustainability. Because of the preliminary evidence for CRM, it is included in several on-going Health Services and Resources Administration projects to increase healthcare workforce resiliency spawning evaluations of their own; these include Children’s Hospital Los Angeles’ Health and Public Safety Workforce Resiliency Training Program (Revitalize), the University of New Mexico ECHO Behavioral Health Provider Workforce Resiliency (BHPWR) program, the University of Alabama Workforce Engagement for Compassionate Advocacy, Resiliency and Empowerment (WE CARE), and Atlanta’s Resiliency Resource for Frontline Workers (ARROW) at Emory University (for links to these programs, see Table 3). CRM has been implemented as part of larger well-being programming efforts as well as stand- alone. In published quality improvement and program evaluation findings, CRM is consistently found to be a feasible and acceptable intervention. However, for these programs, the effects on healthcare worker well-being varied, indicating the need for further study related to implementation such as factors related to context, facilitation, CRM “dose”, and the best measurement approach [98,99,100]. 

In 2015, CRM was integrated into the Social, Emotional, and Ethical Learning program of Emory University, which is now deployed in dozens of countries and is an ongoing research endeavor. Cognitively Based Compassion Training at the Emory University Center for Contemplative Science also incorporates CRM. DBT Skills and CRM have been blended for several years for staff and unhoused youth orientation at Covenant House shelter in Georgia. CRM also integrates well with motivational interviewing [88,101,102,103]. Projects that are in progress currently include teaching CRM to staff, teachers, and 140,000 students and their families in Wake County, North Carolina; 300 incarcerated teenagers in Georgia [102]; and 300 community health workers in 40 Los Angeles County agencies (for links to associated organizations, see Table 3).

### 5.3. Anecdotal Evidence and a Definition of Resilience

CRM was developed at the Trauma Resource Institute (TRI), a non-profit that responds to humanitarian crises. Anecdotal evidence points to CRM’s trans-cultural nature in dozens of countries and its favorable impact in disasters, e.g., the Ukraine war, the Israel–Palestinian conflict, the Paradise Fire, the Las Vegas/Pulse Nightclub Shootings [101], and natural emergencies (see Table 2 for CRM humanitarian efforts). To Miller-Karas, it became evident that regardless of nationality or background, human beings share a commonality in their nervous system responses to both suffering and expressions of hope. Across all these diverse regions where CRM has been implemented, people described their symptoms of traumatic stress in strikingly similar ways. People from diverse cultural and religious backgrounds were able to adapt these skills through their own cultural perspectives, making CRM an accessible and inclusive model. Furthermore, children were observed to grasp and apply these skills just as effectively as adults.

From the many international CRM workshops she has conducted, Miller-Karas has refined a definition of resiliency that encompasses empowerment without negating individual inherent gentleness, vulnerability, and occasional weariness. “Resilience” is a potent term viewed through a strength-oriented lens; its working definition has evolved over the years. Resilience is not a static state but a dynamically emerging force with ebbs and flows. It may seem elusive, like an untapped river of well-being waiting to be explored. It is a multifaceted mosaic of attributes that define resilient individuals and communities. This definition embodies compassion and empathy, acknowledges recent and historical human suffering, and celebrates individual and community assets and strengths, including culture and traditions. Resilience encompasses the embodiment of well-being and emotional regulation. As well-being develops, resilient individuals remain receptive to hope and optimism, displaying adaptability and flexibility in the face of life’s trials. Resilient individuals and communities actively embrace principles of equity, justice, and inclusion [104].

### 5.4. Questions Remain for Interoceptive Awareness Measures

In CRM’s second RCT with healthcare workers during the first year of the pandemic [87], the Noticing subscale of the MAIA-2 was the only part of the scale used because only it fit with the CRM training. Two of the investigators on that study are authors of this paper. Many of the items in the complete MAIA-2 scale [58] do not mesh with CRM’s interpretation of interceptive awareness. For example, the words “feel” or “feeling” are used for many items of the MAIA-2; however, these words are ambiguous, referring to both sensation and emotion. CRM teachers avoid using these words and they emphasize that the skill focus is on sensations, not feelings. Only eight items in the MAIA-2 dove-tail somewhat with the interoceptive awareness as taught in CRM. These are as follows: When I am tense, I notice where the tension is located in my body (item 1); I can maintain awareness of my inner bodily sensations even when there is a lot going on around me (item 17); I can return awareness to my body if I am distracted (item 19); I can refocus my attention from thinking to sensing my body (item 20); I notice how my body changes when I am angry (item 23); I notice that my body feels different after a peaceful experience (item 25); I notice how my body changes when I feel happy/joyful (item 27); and When I feel overwhelmed I can find a calm place inside (item 28). The disconnect between CRM and the MAIA-2 may be that CRM skills focus on noticing and then shifting attention from sensations of discomfort to sensations that are less uncomfortable, neutral, or pleasant. This contrasts with terms used in the MAIA-2 such as powering through or pushing away pain, thoughts, and emotions. A purely CRM item for interoceptive awareness would be, “I notice pleasant, unpleasant or neutral sensations in my body at different times during my day.”

Because of the overlap between interoceptive awareness and mindfulness trainings, mention is also made here of the original 39-item Five Facet Mindfulness Questionnaire (FFMQ) [105], which includes five items that relate to sensation. These are the following: When I’m walking, I deliberately notice the sensations of my body moving (item 1); When I take a shower or bath, I stay alert to the sensations of water on my body (item 6); I notice how foods and drinks affect my thoughts, bodily sensations, and emotions (item 11); I pay attention to sensations, such as the wind in my hair or sun on my face (item 15); and When I have a sensation in my body, it’s difficult for me to describe it because I can’t find the right words (item 22). The FFMQ-15 is a shorter predictor for positive thinking, an overall uplifted mood, and subjective feelings of well-being [106,107] that includes three sensation items.

## 6. Emerging Science Related to Somatic Interventions

### 6.1. Intrapersonal Synchrony

The concepts of “neural synchrony”, as well as synchrony both within (intrapersonal) and between (interpersonal) persons, are explained here. Neural synchrony is the activity and repeated patterns across different brain regions; synchrony is critical for cerebral communication, integration, and information processing. For an individual (intrapersonal synchrony), interoception encompasses the capacity to discern and interpret internal physiological states, incorporating visceral functions, bodily sensations, and affective experiences. The emerging literature underscores an intricate interrelation between neural synchrony and interoception; both synergistically contribute to our awareness of physiological states and affective experiences. Such synchronized patterns within interoceptive networks orchestrate the merging of somatic awareness with cognitive and emotional domains, sculpting our conscious interpretation of somatic sensations and affective modalities [108]. The degree of this orchestrated oscillatory activity within interoceptive circuits (synchrony) appears to be directly proportional to optimal cognitive and emotional function [109].

Recent studies highlight neural synchrony between the insular cortex and other cerebral regions, notably the prefrontal cortex, right temporoparietal junction, cingulate cortex, and inferior frontal gyrus, as pivotal to interoceptive precision and awareness [110,111]. In practicing CRM’s somatic awareness, the essential skill of Tracking is deployed to amplify sensory awareness via intentional interoception, potentially enhancing emotional regulation and fostering a sense of well-being. Gogolla suggests that these domains buttress core capacities such as emotional regulation, empathy, self-concept, and social interactions, with explicit ties to interoceptive processes [22], as is embodied in CRM’s Tracking skill.

The neural synchrony embedded within interoceptive circuits is contingent upon a multitude of variables. Emotive and cognitive paradigms, for instance, possess the potential to recalibrate the amplitude and coherence of neural synchrony inherent to interoceptive pathways [112,113]. Potent emotional stimuli, typified by threats (analogous to the “lightning bolt” in CRM’s dysregulation schema) or rewards, can potentiate the synchronization of cerebral networks, thereby accentuating both euphoric and dysphoric subjective interpretations of physiological and emotional states. The deployment of specific CRM techniques, exemplified by the Help Now! strategy, e.g., naming colors or objects within view, counting, or noticing sensations of standing or stepping forward, demands real-time cognitive engagement, which, in turn, modulates the synchrony of neural activity within the interoceptive circuits.

### 6.2. Interpersonal Synchrony

Interpersonal synchronization refers to the coordinated alignment of behavioral, physiological, hormonal, and neural activities between individuals—such as synchronized gestures, movements, autonomic responses, and neuroendocrine processes like oxytocin release [114]. A related, but more specific construct, interbrain synchronization, describes the temporal coupling of neural activity across individuals and is measurable via neuroimaging methods such as EEG or fMRI hyperscanning. While both forms of synchrony contribute to adaptive social behavior, interpersonal synchronization can be viewed as the broader phenomenon, encompassing the behavioral and physiological dimensions within which interbrain synchronization resides. Research directly linking behavioral and physiological synchrony remains limited, and standardized definitions are still emerging [115]. Although a full review of mechanisms is beyond this article’s scope, this section introduces interpersonal synchronization as a possible explanation for interoception, or more specifically, CRM’s observed effects.

Interpersonal synchronization occurs across multiple biobehavioral tiers, prominently reflected in heart rate variability (HRV), skin conductance response (SCR), and brain electrical activity. It is an adaptive mechanism through which individuals consciously or subconsciously perceive and interpret others’ internal arousal states. For instance, face-to-face interactions significantly amplify physiological synchrony compared to interactions without visual contact, demonstrating the critical role of direct interpersonal feedback [110]. Additionally, hormonal synchrony, particularly involving oxytocin, has been documented as facilitating social bonding and cooperation. Increased oxytocin release between interacting partners not only correlates with stronger emotional bonds but also enhances neural coupling, promoting deeper empathic connections and social attunement [116].

Enhanced synchronization is linked with improved self-regulation, co-regulation, and strengthened feelings of connectedness and empathy [114]. Such synchronization facilitates emotional sharing, expands social connections (e.g., attachments), supports emotional intelligence, and aids adaptation to group behaviors [117]. Within individuals, adaptive synchronization—or conversely, adaptive desynchronization during competition—is essential in responding effectively to stressors associated with states of awareness of oneself, others, and one’s environment [118]. Intentional interoceptive awareness (e.g., Tracking practices in CRM) may significantly enhance this adaptive synchronization process, particularly during disruptions to the Resilient Zone (RZ). Individuals frequently report heightened trust and connectivity during interactions when they are within their Resilient Zone, which could reflect a state-level synchronization that promotes socialization and cooperation. Conversely, deficits in interpersonal synchronization correlate strongly with depression, anxiety, and loneliness—conditions that are increasingly recognized as public health concerns [119,120]. Thus, promoting interoceptive practices in the service of interpersonal synchronization could regulate feelings of isolation and increase social engagement.

At the neurophysiological level, interbrain synchronization allows for individuals to share increased reciprocal neural activity during conversations or shared experiences [121]. Recent hyperscanning studies (using simultaneous functional near-infrared spectroscopy [fNIRS], EEG, or fMRI recordings) have demonstrated robust synchronization in brain regions crucial for social cognition and cooperative engagement, including the prefrontal cortex (PFC), temporoparietal junction (TPJ), superior temporal sulcus (STS), and dorsomedial prefrontal cortex (dmPFC) [122,123]. The temporoparietal junction (TPJ) and superior temporal sulcus (STS) networks, which are integral to social cognition and theory-of-mind processing, show pronounced synchronization during joint activities requiring mutual understanding and prediction of others’ actions. In addition, neural synchronization within frontal and parietal regions facilitates the alignment of attentional and predictive cognitive processes between interacting individuals [108,124].

When individuals have shared goals or are cooperating, their social interactions can induce a phenomenon known as attunement, synchronization, or social alignment [117]. These neural synchronizations are linked with cognitive processes involving mental modeling, predictive inference, emotional dynamics, and risk–reward assessments [125]. In a recent systematic review and meta-analysis by Czeszumski and colleagues (2022) [122], thirteen studies (N = 890) identified the PFC as central to temporoparietal interbrain synchronization during cooperative engagements. Significant interbrain coupling in the PFC and TPJ (including the so-called mirror neuron system) has consistently emerged during cooperative tasks, underscoring their role in facilitating joint attention, shared intentionality, and adaptive social responses [126].

Repeated social interactions can induce lasting changes in interbrain functional connectivity, supporting Hebbian principles—”neurons that fire together, wire together” [117]. Neural synchronization mechanisms involve oscillatory coupling, where brain waves align in frequency and phase between interacting individuals, particularly notable in beta-band oscillations (~13–30 Hz) associated with joint tasks [127]. This neural synchrony appears to reflect a genuine integrated hyper-brain network, wherein neural activity patterns across multiple brains become temporally and functionally coupled, enabling effective social interaction and cognitive alignment [117]. Such synchronization is thought to be governed by mesolimbic neurotransmitter systems (including anterior insula and anterior cingulate cortex) operating through reward-feedback loops, sensorimotor coordination, modulating selective attention, and emotional engagement. Extending from this, it is possible optimal interpersonal synchronization involves access to the RZ or the brain’s “thinking networks”.

In educational and caregiving contexts, synchronized neural and physiological activities (e.g., brain waves) correlate positively with learning outcomes, cooperative success, emotional resilience, and overall well-being [128,129]. Recent randomized controlled trials have further illustrated that interventions like virtual CRM training could enhance interpersonal synchrony, resulting in improved perceptions of teamwork and relational dynamics among healthcare providers [87]. Consequently, care providers employing intentional emotional and somatic regulatory practices can effectively influence others’ perceptions, maintain or restore access to the RZ, and foster adaptive interpersonal synchrony.

## 7. Discussion

### 7.1. A Sensory Awareness Connects to Health

Michel de Montaigne, a Renaissance philosopher, may have been the first person to try to communicate the concept of paying attention to body sensation. Because of a near-death experience around 1570, he experienced “floating sensations, the feeling of his breath or spirit, lingering at the threshold of his body” [130] (p. 32). As a result, he acquired a new sense of being alive and subsequently focused on sensations, not for what they were supposed to mean, but the way they felt. Sensory awareness taught him not to worry about death; in fact, by paying attention in the moment, he had a sense of immortality. He observed the world more closely, and in his writing, Montaigne described inner sensations and social encounters with precision [130].

Fast-forwarding to today, there has been a relatively recent convergence of movements and research that has advanced thought on resiliency, recovery from trauma, and self-care agency that form a paradigm shift. These include work in the fields of ACEs, trauma-informed care, positive psychology, post-traumatic growth, mindfulness, the neurobiology of interoception, body maps of emotions, body-based psychotherapy, self-care, motivational interviewing, energy psychology, and the inception of mind–body institutes at major universities, among other evidence of change and progress. Whether via routes originating in meditation and mindfulness or via routes emanating from trauma biology and body psychotherapy, there appears to be an increased awareness that states that induce mind–body connection promote health, well-being, and emotion regulation.

### 7.2. Clarifying the Difference Between CRM and Similar Interventions

During mindfulness-based interventions such as Mindfulness-Based Stress Reduction (MBSR) and Mindfulness-Based Cognitive Therapy (MBCT), focused attention on present-moment sensations can inadvertently activate unresolved traumatic material, potentially resulting in heightened distress or dysregulation. The Community Resiliency Model (CRM) supports individual agency by offering structured, somatically based skills that invite individuals to intentionally shift their attention toward sensations associated with well-being and safety. This emphasis on choice and self-regulation reflects CRM’s trauma-informed framework, prioritizing autonomy and empowerment in the healing process. Moreover, unlike MBSR and MBCT, which often require a designated quiet space for practice, CRM skills are designed for flexible integration into the flow of daily life, making them particularly accessible in diverse and high-demand settings. 

Mindfulness practice is integrated throughout DBT Skills Training and includes some sensory awareness techniques for emotion regulation and distress tolerance. The concept of “Wise Mind” (a mix of emotion and reasoning brain states) is somewhat akin to being in the Resiliency Zone (RZ); however, CRM teaching expands the concept with a neurobiological explanation for shifts in the RZ and what happens when we are knocked out, which explains some of our common mental health states. CRM concepts are simple and easy to grasp, without complex nomenclature, and CRM skills are mainly focused on simple sensory awareness techniques to attain distress tolerance and emotion regulation. 

The Trauma Resiliency Model (TRM) is designed for use by licensed mental health professionals within clinical practice settings. While it incorporates the wellness skills foundational to the Community Resiliency Model (CRM), TRM emphasizes the reprocessing of traumatic experiences under the guidance of a trained TRM practitioner. In contrast, the Community Resiliency Model Teacher Training Program prepares both natural community leaders and mental health professionals to disseminate CRM as a biologically based wellness approach. CRM may be utilized as a self-care practice or shared within individual and group settings, and, importantly, does not require facilitation by a licensed mental health professional, thereby increasing accessibility and scalability across diverse populations. 

Both the Community Resiliency Model (CRM) and Somatic Experiencing (SE) are somatically based, neuroscience-informed approaches that support nervous system regulation in the aftermath of trauma and chronic stress. CRM is a trauma-informed wellness model designed to increase individual and community resilience through six core self-regulation skills. It emphasizes stabilization, preventing re-traumatization, and accessibility, allowing trained community members—including non-clinicians—to disseminate the model in diverse settings such as schools, disaster response, and healthcare systems. In contrast, SE is a clinical modality focused on resolving trauma by facilitating the completion of the body’s innate survival responses (fight, flight, freeze). It engages clients in titrated somatic awareness and is intended for use by licensed mental health or somatic professionals in therapeutic contexts. CRM does not involve direct trauma processing and is designed for brief training and broad dissemination as a community-based wellness practice. SE, by contrast, requires multi-year training and clinical expertise to guide individuals through the processing and discharge of unresolved traumatic material.

### 7.3. The Value of CRM from a Public Health Perspective

**Figure 3:**
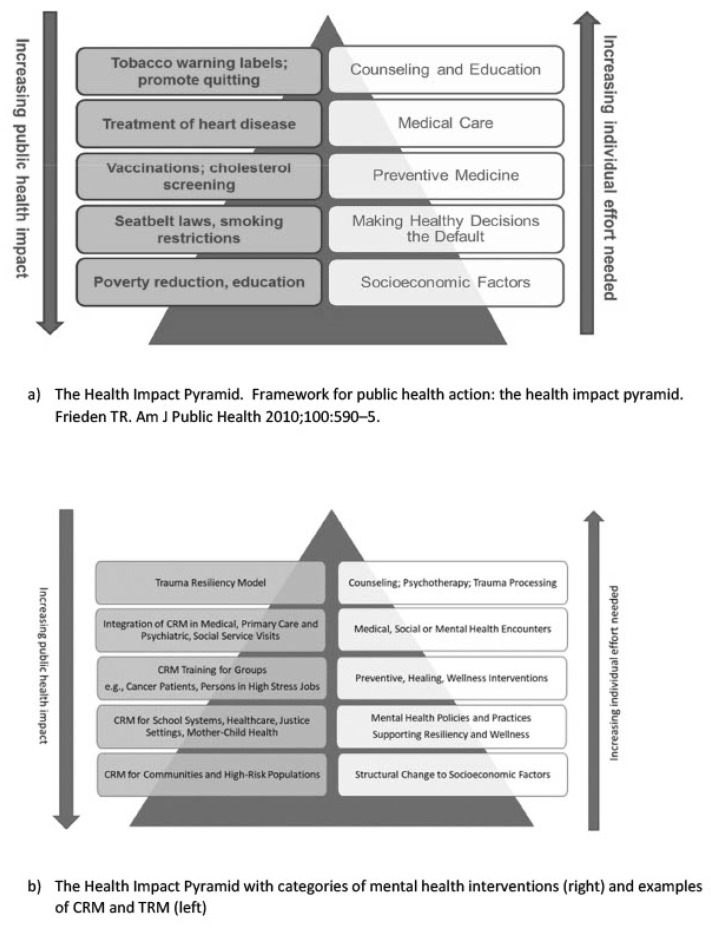
CRM aligned with Thomas Frieden’s Health Impact Pyramid [131].

Brief interoceptive awareness interventions can be contextualized within two public health frameworks: the Health Impact Pyramid (HIP) and the task-shifting (or sharing) approach in healthcare delivery, offering insight into their potential scalability and reach. Thomas Frieden’s Health Impact Pyramid is a schema for categorizing healthcare interventions (Figure 3) [131]. The uppermost levels of the pyramid represent the time-intensive, expensive, one-to-one interventions, like psychotherapy or physician visits; the impact on the individual may be great, but the general population effect is low. The lower-tier interventions of the pyramid include systemic prevention efforts with a far greater impact on populations, but these efforts require societal commitment to changing the social determinants of mental health. Population-level primary prevention to promote mental well-being and reduce ACEs and other risk factors for mental illness, addiction, violence, and illness is situated at the HIP base. Training in interoceptive awareness, e.g., CRM, is a middle-level intervention in the HIP, i.e., a brief or one-time intervention that may confer long-range protective benefits. U.S. health spending is primarily located at the top two layers of the pyramid [132], highlighting the lack of tools to offer psychological help to many people. The HIP reminds us that we should reach broader audiences to improve general mental health, and this brings us to consider how simple body awareness techniques might be disseminated broadly.

CRM, as a self-care somatic awareness training, can also be added to the tool-box of “task-sharing” modalities in healthcare. Non-behavioral health providers can offer therapeutic help to persons experiencing mental distress [133,134]. Mental Health First Aid, referenced above, also falls into this category of intervention. Bottlenecks impeding access are decreased with “Task-sharing” because non-behavioral health specialists can share knowledge and skills. A more collaborative practice and appropriate delegation recruits lower-level healthcare workers to deliver services, which is critical for poorly resourced areas. Extending this approach to lay persons or community members can further extend access and address the large treatment gaps that exist, particularly in poorly resourced countries or even in wealthy countries with healthcare inequities, such as the U.S. CRM is naturally positioned as a task-sharing model. It is taught formally by certified teachers who may be community members, yet any person who understands CRM skills and concepts can share them with others informally as a “CRM Guide”. Guided dissemination scales CRM to a higher level of reach and impact and is comparatively inexpensive. Its foremost utility lies in how quickly the skills can be learned. For some, CRM only requires a small “dose”. Measurable effects on distress and well-being were found after just a single 1 h CRM class, requiring only those resources naturally carried within the body.

## 8. Future Research Directions

### 8.1. Brief Skills Training Knowledge Gaps

The demand for more research on the role of interoception in mental health is coming from many directions: psychiatry, psychology, neurology, neuroscience, and physiology. Work is already occurring in these fields on interoceptive assessment, interoceptive integration, and interoceptive psychopathology [50,51,52,53]. Because of the potential population impact of brief interoceptive awareness interventions that can impact emotion regulation and mind–body synchrony, programs that are simple and easy to disseminate (like CRM) must find a space within these research contexts and sectors.

Although body awareness skills may be valuable for all persons, we would like to encourage future applications of CRM to those with emotion dysregulation, substance use, pain, mental health symptoms/disorders, and the other downstream effects of ACEs. In addition, access to wellness skills may be beneficial to members of marginalized groups as a means of coping with adversity and/or loneliness. Marginalized groups can include persons who are incarcerated or justice-involved, LGBTQAI+, unhoused, indigenous, minority, intellectually disabled, economically disadvantaged, refugees, domestic violence survivors, elderly, and persons who have serious mental illness or are members of other underserved groups.

Suggested research for brief interoceptive awareness skill training might include the following:

(1) Biobehavioral measures of emotion regulation (cortisol levels, heart rate variability, electroencephalography, eye tracking);
(2) Self-report or observation data on specific mental health problems (e.g., anxiety, depression) or behaviors (e.g., substance use, violence, incarceration);
(3) Positive mental health parameters: well-being, emotion regulation, resiliency, self-care;
(4) Pro-social thinking and behaviors such as teamwork, communication, empathy, and self- and other-compassion;
(5) Perception of the self, other, and the environmental (SOE) system using technically supported biobehavioral measures;
(6) Interpersonal synchronization of the SOE and its impact on work and learning, such as in groups and classrooms;
(7) Community or public health outcomes;
(8) Qualitative research on special groups, e.g., mother–infant interaction and family experience;
(9) Brain imaging to detect the development of neural pathways and networks that reflect post-traumatic growth or resiliency pathways that counteract the harm of ACEs and trauma.

Some research questions specific to CRM are the following: (1) What are the different impacts between CRM and mindfulness, or other interoception trainings? (2) How do CRM skills and concepts differ from other interoceptive awareness programs? (3) What are the minimum doses for teaching CRM for different contexts or audiences? (4) What measures are best suited to reflect changes in mental health and resiliency associated with a CRM training? (5) How can CRM be integrated into successful models that are already in place?

### 8.2. Hypotheses for Consideration in Brief Interoceptive Awareness Training Research

Given the clear emerging evidence supporting a simple interoceptive awareness training in somatic self-care habits, a wide-angle lens for future research might consider the following hypotheses:

1. Individuals who use somatic skill training will experience (a) greater self- and other compassion; emotion regulation; empathy; sense of well-being, and (b) reduced symptoms of stress, anxiety, depression, substance use, and other mental health issues.
2. Individuals who suffer from chronic illness and who practice somatic skills will demonstrate a reduction in psychological distress and possibly markers of their illnesses.
3. Members in group settings (e.g., in classrooms, day programs, community groups) who acquire somatic skill training will exhibit intra- and inter-brain synchrony as measured by heart rate variability, electroencephalography, and other biologic parameters.
4. Communities that disseminate somatic skill trainings widely will have lower rates of the disorders and patterns identified as ACE outcomes, e.g., violence, substance use disorders, and mental health problems, e.g., suicidality, depression, loneliness, anxiety, PTSD, drug overdose.
5. Communities that have suffered trauma (fires, shootings, natural disasters) that receive somatic skill interventions will have lower than expected subsequent rates of PTSD and other mental health fallout. Likewise, communities that are trained in somatic awareness *prior to* human-made or natural disasters will better withstand the impact of these events and will have a greater capacity to engage in mutual and community support within those disaster settings.

## 9. Conclusions

Good sleep, healthy eating, exercise, and positive relationships are at the crux of well-being, but such good habits may be elusive for persons who have survived trauma. The numbers of people impacted by traumatic events (most people) far exceed the mental health resources available to help them. Even if there were enough behavioral health providers, two major barriers exist: (1) many people are averse to seeking mental health care due to its stigma, and (2) often such help is out of reach financially, geographically, or practically. Therefore, a public health perspective is needed to identify easily disseminated modalities that can be scaled up for existing global needs. CRM has emerged as a stand-alone, preventative intervention—a kind of mental health self-care—to be adapted for local needs and blended with other interventions, with a compassionate perspective on mental health. The existence of other brief interoceptive awareness programs and mindfulness programs is clearly a move forward. CRM is an example of an interoceptive training that can serve as a feasible framework for reducing the impact of trauma on mental health. Human beings will continue to experience trauma, but as a resilient species, we can adopt somatic awareness techniques to care for ourselves and positively adapt, therefore reducing the burden of disease that trauma brings.

The neurophysiology of trauma and resilience is currently under intense investigation and presents a promising opportunity to find ways to cope with stress, crisis, mental illness, and trauma. We can begin to work with our bodies to achieve greater internal harmony, and our bodies and brains can then become more accessible and malleable to heal from trauma and ACEs. We all have built-in mechanisms for self-care to achieve greater well-being, but we need to know how to access them. We hope this article has illuminated some of the mechanisms of action of sensory awareness self-care (1) for use immediately in a crisis, by interrupting loss of executive override, (2) for its longer-term impact on positive mental health variables and a wider Resilient Zone, and (3) for its possible impact on pro-social behavior and synchronization to optimize social cohesion and learning.

Antonio Damasio stated that the core self is the sum of extero- and interoceptive inputs that form the experience of the self as an integrated entity [16,135]. If human beings can sense the energy of their best, whole selves, they can live more vibrantly and better manage challenges in their lives, including getting along with one another. If disseminated in communities globally, somatic awareness skills such as CRM may change the score the body keeps for many, many people.

## Figures and Tables

**Table 1:** The six wellness skills of the Community Resiliency Model.

	Skill	Examples
1.	**Tracking:** Monitoring one’s own physical sensations, both external and internal.	While walking, I focus on the strength in my legs; in difficult situations, I can “sense-in” to my body, noticing sensations in my chest, abdomen, or limbs.
2.	**Resourcing:** Identifying resources, including internal (e.g., a personal strength or characteristic), external (e.g., faith, family, friends, pets), or imagined (e.g., a superhero, a literary character) helps individuals return to a state of balance during or after a stressful experience.	I recall a beach scene as a child, remembering the salty smell, the crash of surf, the heat of the sun, the sand sticking to my arms. When I think about the scene, I take a deep breath and notice stability in my chest.
3.	**Grounding:** Paying attention to the body or part of the body making contact with a surface of support, which can be calming and help people to be present in the moment.	I bring attention to the sensation of pavement under my feet on my way to the parking deck. I notice the texture and temperature of my steering wheel while driving, my clothing, my own skin, settling me.
4.	**Gesturing:** Gestures can emerge spontaneously, usually below conscious awareness, like a self-soothing touch. Gesturing involves intentionally making a movement or gesture associated with well-being, e.g., joy, happiness, courage.	When I am stressed, I put my hand over my chest or rub my wrist or knuckles. I notice I take a deeper breath at that moment and feel lighter.
5.	**Help Now!:** Inducing a resiliency pause, ten sample strategies are taught that can reset the nervous system when a person is in a hyper- or hypo-aroused state to help them return to a state of balance.	When I feel overwhelmed, I name the colors or objects in the room to myself, noticing a calming as I proceed. I can push my arms against a wall, engaging the muscles. Sensations of energy move from my chest out to my arms, I am less distressed.
6.	**Shift and Stay:** Discerning whether we are in a balanced state or not while Tracking, intentionally shifting or staying with neutral or pleasant sensations for about 10–15 s to widen the Resilient Zone.	I was in a bad mood and thought of my beach resource, remembering the sensory details of that experience. I stayed thinking about it and noticing sensations for about 15 s and noticed relief and balance.

Note: This table was adapted from a prior publication [73].

**Table 2:** Community Resiliency Model disaster responses.

China Sichuan Province Earthquake	2008–2010
Haiti Earthquake	2010–present
Philippines Typhoon Haiyan	2014–2020
Rwanda (post-genocide trauma)	2009–present
Nepal Katmandu Earthquake	2015–2016
Northern Uganda War- Lord’s Resistance Army	2016–present
Mass Shootings: San Bernardino, CA, Pulse Attack, FL, Dayton, OH, Thousand Oaks, CA, Nashville, TN.	2015–present
Butte County, CA (Camp Fire)	2019–2020
Ukrainian Humanitarian Resiliency Project	2022–present
Maui, Hawaii (in process)	2023–2024
Belfast, Northern Ireland	2016–present
Turkey (Syrian Refugees)	2016–1017
Angola, Post Civil War Project, Resiliency Project	2023–present
Israel Humanitarian Project	2023–present
Palestine Humanitarian Project	2023–present

**Table 3:** Links to programs and organizations using CRM in trainings.

The Rwanda Resilience and Grounding Organization	https://www.rrgo.org/ (accessed on 10 May 2025).
Social, Emotional and Ethical Learning program of Emory University	https://compassion.emory.edu/see-learning/ (accessed on 10 May 2025).
Cognitively Based Compassion Training at the Emory University Center for Contemplative Science	https://compassion.emory.edu/cbct-compassion-training/index.html (accessed on 10 May 2025).
Trauma Resource Institute (TRI)	https://www.traumaresourceinstitute.com (accessed on 10 May 2025).
Health Resources and Services Administration Grantees 2022–2024:	https://bhw.hrsa.gov/funding/health-workforce-resiliency-awards (accessed on 10 May 2025).
Emory University-Atlanta’s Resiliency Resource fOR frontline Workers (ARROW)	https://www.nursing.emory.edu/initiatives/arrow (accessed on 10 May 2025).
Children’s Hospital of Los Angeles Health and Public Safety Workforce Resiliency Training Program (Revitalize)	https://www.chla.org/blog/experts/work-matters/chlas-revitalize-program-helping-team-members-preserve-their-mental-health (accessed on 10 May 2025).
The University of New Mexico ECHO Behavioral Health Provider Workforce Resiliency (BHPWR) program	https://iecho.org/echo-institute-programs/supporting-resilience-through-health-care (accessed on 10 May 2025).
University of Alabama Workforce Engagement for Compassionate Advocacy, Resiliency and Empowerment (WE CARE)	https://www.uabmedicine.org/news/we-care-focuses-on-well-being/ (accessed on 10 May 2025).
Convent House Georgia wellness programming	https://covenanthousega.org/Health-Wellness-Services (accessed on 10 May 2025).
Atlanta Birth Center “Wellness Within” program	https://www.atlantabirthcenter.org/ (accessed on 10 May 2025).
Wake County Public School System, Counseling and Student Services Wellness workshops	Various sites such as: https://www.wakeahec.org/courses-and-events/72134/lets-come-together-lets-crm-together-community-resiliency-model (accessed on 10 May 2025).
